# Cultivation System Dominates Cucumber Performance and Root-Zone Microbiomes Across Biochar Particle Sizes

**DOI:** 10.3390/plants15111627

**Published:** 2026-05-26

**Authors:** Seyed Mohammad Hashemi, Moritz Graeff, Emmanuel A. Nai, Nick Savidov

**Affiliations:** 1 The Centre for Applied Research, Innovation and Entrepreneurship, Lethbridge Polytechnic, Lethbridge, AB T1K 1L6, Canada; emmanuel.nai@lethpolytech.ca; 2 Integrated Agriculture Technology Centre, Lethbridge Polytechnic, Lethbridge, AB T1K 1L6, Canada; 3 Vivent SA, Rue Mauverney 28, 1196 Gland, Switzerland; moritz.graeff@vivent.ch

**Keywords:** aquaponics, hydroponics, biochar particle size, cucumber, nutrient dynamics, electrophysiology, root-zone microbiome

## Abstract

Hydroponic (HP) and aquaponic (AQ) systems are widely known in greenhouse production; however, the combined effects of nutrient delivery system and substrate physical structure on crop performance and root-zone microbiomes remain insufficiently understood. Substrate physical properties influence water retention and aeration, which can affect root-associated microorganisms, plant growth, and yield. This study evaluated cucumber (*Cucumis sativus* L.) growth, yield, nutrient dynamics, physiological stress responses, and bacterial community composition under HP and AQ systems using bamboo-derived biochar substrates and coconut coir as a control. Vegetative growth was enhanced under AQ, with the greatest plant elongation (1102 ± 40.1 cm) and stem diameter (15.1 ± 1.0 mm) observed in biochar-grown plants. Total yield was consistently higher under AQ than HP, with the highest yield recorded in the coarse biochar treatment (28.6 kg m^−2^). Aquaponic systems were associated with greater nutrient availability under the conditions evaluated during mid to late season production, including nitrate concentrations of up to 226 mg L^−1^. Physiological stress monitoring indicated lower stress exposure under aquaponic conditions in plants grown in medium and coarse biochar substrates across both systems, with 78 to 81% of the growing season classified within low to balanced stress conditions. Bacterial community composition was primarily shaped by cultivation system, which explained 19.3% of the observed variation, whereas substrate treatment did not significantly alter overall bacterial community structure. Overall, cultivation system was the dominant factor associated with variation in cucumber performance and root-zone bacterial communities, while biochar substrates supported improved plant growth, yield, and reduced physiological stress.

## 1. Introduction

Hydroponics (HP) is among the most widely adopted Controlled Environment Agriculture (CEA) cultivation systems for intensive vegetable production, offering resource-efficient alternatives to conventional farming [1,2]. Aquaponics (AQ) is an emerging technology to produce food in a sustainable manner based on an ecosystem approach, gaining popularity worldwide [3,4,5]. HP relies on mineral nutrient solutions that provide growers with a high degree of control and predictability, while AQ integrates aquaculture effluents into plant cultivation, creating a biologically enriched, circular nutrient cycle where beneficial microorganisms play a pivotal role [6,7,8,9]. These systems differ in microbial diversity and resilience, traits that strongly influence plant performance [1,5,10]. Understanding these differences is essential for optimizing plant physiology, yield, and root-zone ecology to achieve both sustainability and commercial scalability in CEA.

Within media-based HP and AQ systems, substrate choice is another critical factor shaping water retention, nutrient buffering, and microbial colonization [11]. Coconut coir and rockwool substrates are widely used commercially, but biochar, a porous, carbon-rich material produced through biomass pyrolysis, has emerged as a promising sustainable alternative in soilless systems [12,13,14]. Early studies at the Crop Diversification Centre South, Alberta, Canada, highlighted its stability, porosity, and water holding capacity for soilless vegetable production [15,16]. Building on these findings, researchers developed biochar-based growing media for both hydroponic and aquaponic systems using a range of feedstocks, including straw, woodchips, sawdust, rice husks, and coconut coir [17,18,19]. These studies demonstrated the versatility of biochar as a sustainable alternative to conventional soilless substrates. In addition, its high cation exchange capacity and surface area make it suitable for nutrient retention and microbial interactions, while aligning with sustainable bioeconomy principles [20,21,22]. Nevertheless, the performance of biochar is not uniform; its physical properties, particularly particle size, can alter porosity, oxygen diffusion, and water-holding capacity, with potential consequences for plant growth and root-associated microbiomes [13,23,24].

Although HP and AQ are both recognized as sustainable cultivation systems, most comparative studies have focused on yield and nutrient cycling [1,2,3,4,5,6], with few integrating plant physiology, stress responses, and root-zone microbial ecology in a single framework. Similarly, although the role of substrates has been reviewed [8,9] and biochar has been shown to enhance crop productivity and nutrient retention [25], its particle-size effects have not been systematically assessed across different cultivation systems. Each of these factors, cultivation system and substrate properties, has been examined individually, but their combined influence on crop performance and root-zone ecology in CEA remains unexplored.

To address this, we conducted an experiment to investigate the influence of biochar particle size on cucumber (*Cucumis sativus* L.) performance under both hydroponic and aquaponic conditions. Three distinct grades of bamboo-derived biochar (fine, medium, and coarse) were tested and compared with coconut coir as a commercial control. Key response variables included plant growth, fruit yield and quality, nutrient dynamics, stress physiology, and microbial community composition. This study provides a comprehensive evaluation of how cultivation systems and substrate particle size together influence crop productivity and root-zone ecology in CEA.

## 2. Results

### 2.1. Plant Growth and Morphological Traits

Cucumber plant morphology was influenced by both cultivation systems and growing medium (Table 1). Overall, aquaponic treatments outperformed hydroponics in terms of stem elongation and internode distance. The tallest plants were observed in aquaponics with medium-size bamboo biochar (1102.0 ± 40.1 cm), followed by fine and coarse biochar treatments, whereas the shortest plants were recorded under hydroponics with coconut coir (985.2 ± 35.4 cm). No significant differences in stem diameter were observed among treatments. Internode distance followed a similar trend, with aquaponics combined with medium biochar producing the longest internodes (10.6 ± 0.6 cm), and hydroponic coir resulting in the shortest (9.4 ± 0.5 cm).

### 2.2. Yield and Fruit Quality

**Figure 1:**
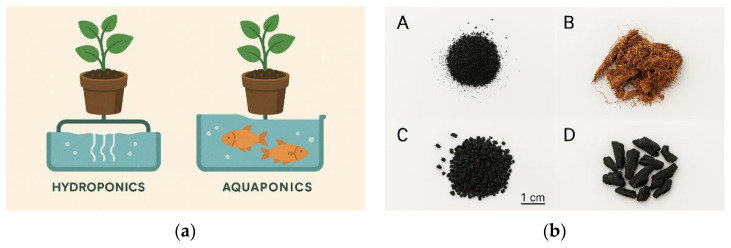
Cultivation systems and substrates used in the experiment. (**a**) Representative cultivation systems, including hydroponic and aquaponic systems. (**b**) Representative substrate materials and particle-size classes: (**A**) fine-grade biochar (1–3 mm), (**B**) coconut coir control, (**C**) medium-grade biochar (3–6 mm), and (**D**) coarse-grade biochar (6–10 mm). Scale bar = 1 cm and applies to all panels.

**Figure 2:**
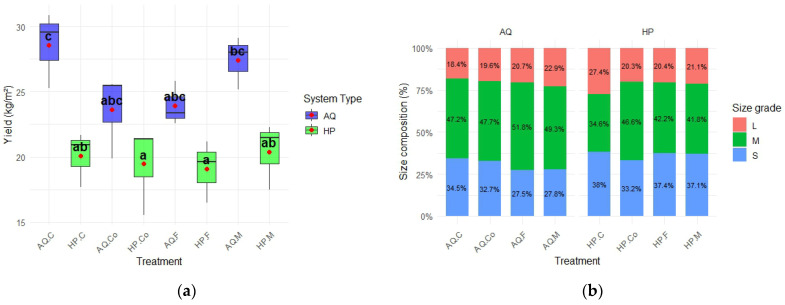
Yield and fruit size distribution of cucumber under different cultivation systems, aquaponics (AQ) and hydroponics (HP), and growing media (C = coarse, Co = coconut coir, F = fine, M = medium). (**a**) Boxplots represent yield (kg m^−2^), with means shown as red points. Different lowercase letters indicate significant differences among treatments according to Tukey’s HSD test (*p* < 0.05). (**b**) Proportional distribution of fruit size grades (L = large, M = medium, S = small) across treatments.

Yield was significantly influenced by both the cultivation systems (HP and AQ) and the type of growing medium (Table 1; Figure 1). Across treatments, AQ systems consistently produced higher cucumber yields compared with HP systems (Figure 2). Total yield differed among treatments, with consistently higher yields observed under AQ conditions compared with HP (Figure 2a). Estimated marginal mean yields under AQ ranged from 23.6 to 28.6 kg m^−2^, whereas HP treatments produced 19.1 to 20.4 kg m^−2^. The highest yield was recorded under AQ with coarse biochar (28.6 kg m^−2^), followed by AQ medium biochar (27.4 kg m^−2^). Across matched substrates, AQ treatments consistently outperformed HPs, while yield differences among HP substrates were comparatively small.

Fruit size distribution varied between cultivation systems and substrates (Figure 2b). AQ treatments produced a higher proportion of medium-size fruits (47.2–51.8%) and a lower proportion of small fruits (27.5–34.5%) compared with HP treatments, which showed 41.8–46.6% medium size and 33.2–38.0% small fruits. Large fruits accounted for 18.4–22.9% of production under AQ and 20.3–27.4% under HP, with the greatest proportion recorded under HP course-size biochar (27.4%). Overall, AQ systems favored a shift toward medium-size fruit production, whereas HP systems produced a higher proportion of small fruits.

### 2.3. Nutrient Dynamics

**Figure 3:**
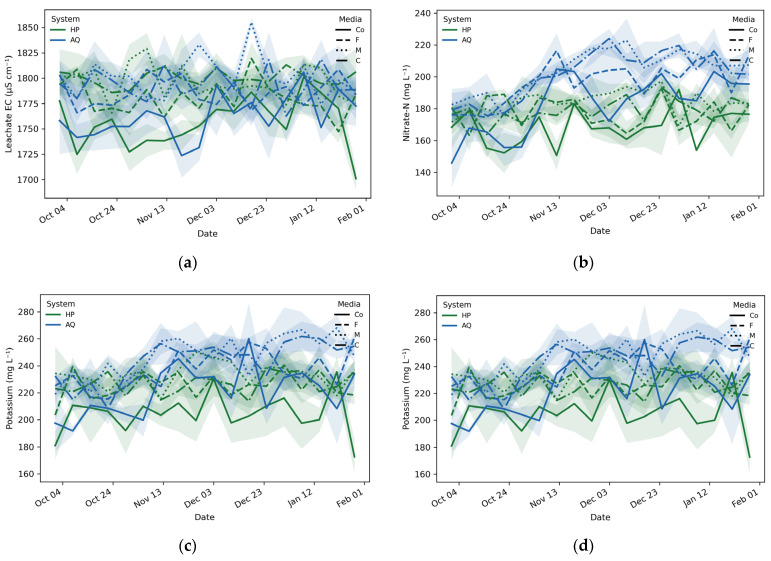
Seasonal dynamics of leachate electrical conductivity (EC) and nutrient concentrations in cucumber (*Cucumis sativus* L.) grown under hydroponic (HP) and aquaponic (AQ) systems. Panels show (**a**) electrical conductivity (EC), (**b**) nitrate-N (NO_3_–N), (**c**) potassium (K), and (**d**) calcium (Ca) over the growing period. Lines represent treatment means, and shaded ribbons indicate ± standard deviation (SD). Line color denotes cultivation system (HP vs. AQ), while line style represents growing media (coconut coir, fine biochar, medium biochar, and coarse biochar).

Across all treatments, EC values were maintained within a narrow range of approximately 1720 to 1860 µS cm^−1^, with no consistent separation between systems or growing media (Figure 3a). In contrast, leachate nutrient concentrations exhibited clear temporal and system-dependent divergence (Figure 3b–d). Leachate NO_3_–N concentrations in HP treatments ranged from 160 to 185 mg L^−1^, while AQ treatments were associated with higher concentrations (Figure 3b). Potassium concentrations followed a similar pattern, with HP values typically between 200 to 235 mg L^−1^, compared with 235 to 270 mg L^−1^ in AQ treatments, particularly in medium and coarse biochar substrates (Figure 3c). Calcium concentrations also diverged over time, with AQ systems reaching 170 to 190 mg L^−1^, compared with 140 to 165 mg L^−1^ in HP systems (Figure 3d). Growing media influenced leachate nutrient behavior within both systems. Medium and coarse biochar treatments generally exhibited higher and more stable concentrations of NO_3_–N, K, and Ca compared with coconut coir, while fine biochar showed intermediate responses. Variability in leachate nutrient concentrations, reflected by wider standard error bands, was greater in AQ systems than HP systems, particularly during mid-season (Figure 3b–d).

**Figure 4:**
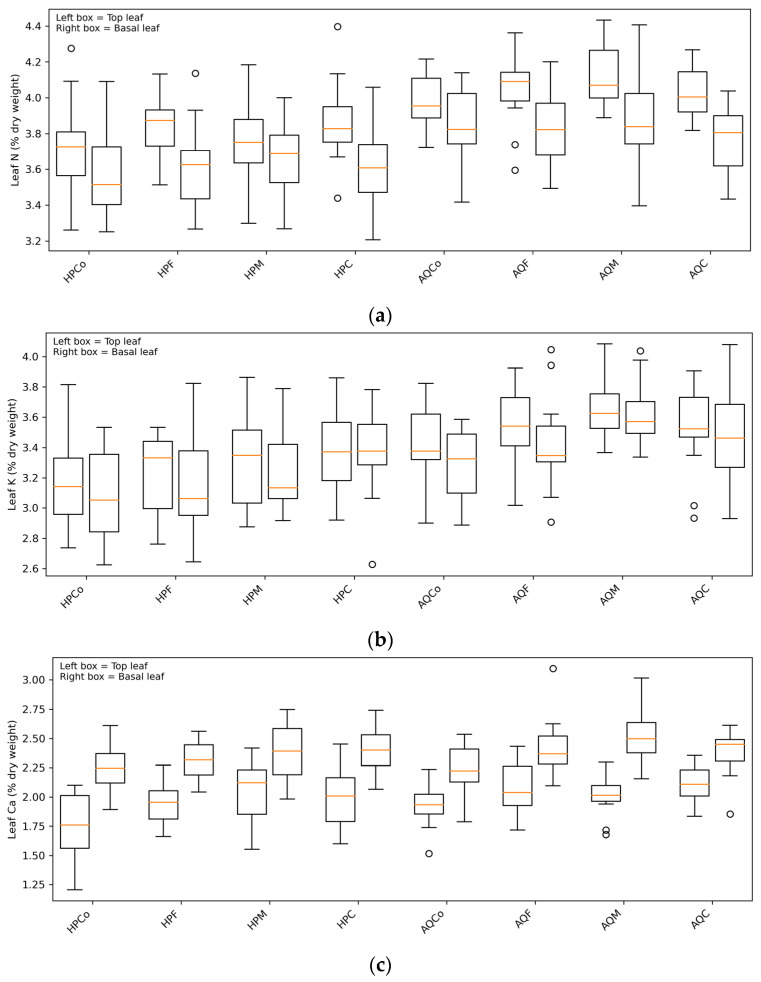
Leaf tissue nutrient concentrations of cucumber (*Cucumis sativus* L.) grown under hydroponic (HP) and aquaponic (AQ) systems with different growing media. Panels show (**a**) nitrogen (N), (**b**) potassium (K), and (**c**) calcium (Ca), expressed as percentage of dry weight. For each treatment, paired boxplots represent nutrient concentrations in the top fully expanded leaves (left box) and basal leaves (right box), as indicated in each panel. Boxes represent the interquartile range with median values shown as horizontal lines; whiskers indicate data dispersion and points denote outliers. Statistical groupings are provided in Table S5.

Leaf tissue nutrient concentrations reflect the net accumulation and redistribution of nutrients within plant tissues over the growing period (Figure 4; Table S5). Leaf nitrogen (N) concentrations ranged from 3.60 to 4.07% dry weight across treatments. In HP treatments, top-leaf N concentrations generally ranged from 3.70 to 3.88%, whereas AQ treatments showed higher values, particularly in biochar-grown plants, reaching up to 4.07%. Basal leaves generally contained lower N concentrations than top leaves across treatments (Figure 4a; Table S5).

Leaf potassium (K) concentrations ranged from 3.07 to 3.62% dry weight across treatments. AQ treatments generally exhibited higher K concentrations than HP treatments, particularly in medium and coarse biochar treatments. Differences between top and basal leaves were smaller for K than for N but remained generally consistent across treatments (Figure 4b; Table S5).

Leaf calcium (Ca) concentrations generally ranged from approximately 1.8 to 2.5% dry weight across treatments. Basal leaves consistently accumulated higher Ca concentrations than top leaves, and AQ treatments, particularly those with medium and coarse biochar, showed the highest Ca accumulation (Figure 4c; Table S5).

### 2.4. Plant Stress Response

**Figure 5:**
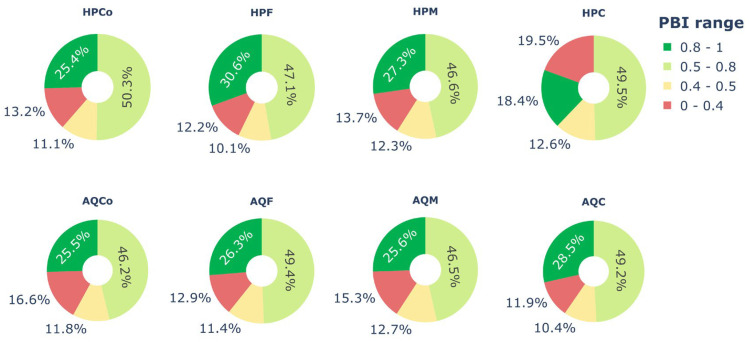
Distribution of Plant Balance Index (PBI) values across hydroponic (HP) and aquaponic (AQ) systems and growing media treatments (Co = coconut coir; F = fine biochar; M = medium biochar; C = coarse biochar). Donut charts represent the proportion (%) of observations within four PBI ranges: 0.8–1.0 (low stress), 0.5–0.8 (moderate balance), 0.4–0.5 (mild stress), and 0–0.4 (high stress).

Clear differences in stress distribution were observed among cultivation systems and growing media (Figure 5). In the HP system, plants grown in coconut coir and fine biochar experienced higher stress exposure. Specifically, plants grown in coconut coir spent approximately 14% of the growing season under high stress (PBI < 0.4), while those in fine biochar exhibited 13 to 14% of the season under mild stress conditions. In contrast, medium and coarse biochar substrates under HP showed improved stress balance, with more than 50% of the growing season spent in the moderate balance range and reduced exposure to low PBI values (<12%).

Under AQ conditions, overall stress exposure was lower across all substrates. Plants grown in medium and coarse biochar under AQ exhibited the most favorable stress profiles, spending 48 to 53% of the season in the moderate balance range and approximately 25 to 30% in the low-stress range. Notably, plants grown under AQ with medium biochar showed the highest physiological stability, with 29.7% of the growing season in the low stress range, while time spent under high stress remained low across all AQ treatments (<11% of the season).

### 2.5. Microbial Community Composition

**Figure 6:**
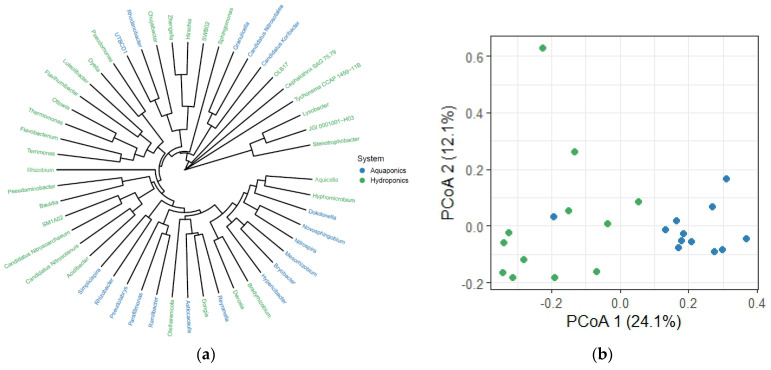
Genus-level microbial community structure under aquaponic and hydroponic systems. (**a**) Dendrogram of the top 50 bacterial genera based on Bray–Curtis dissimilarity of relative abundances, with tip colors indicating the dominant production system (aquaponics or hydroponics) for each genus. (**b**) Principal coordinates analysis (PCoA) of Bray–Curtis distances showing separation of aquaponic and hydroponic samples at the genus level.

Across all samples, a diverse bacterial community was detected, with the 50 most abundant genera accounting for the majority of total relative abundance across systems. Hierarchical clustering based on Bray–Curtis dissimilarity revealed a clear system-level structuring of microbial communities, with AQ and HP samples forming distinct clusters (Figure 6a). This pattern was further supported by principal coordinate analysis, which showed separation of samples along the primary ordination axes according to production system (Figure 6b).

PERMANOVA confirmed a significant effect of production system on microbial community composition, explaining 19.3% of the total variation. In contrast, growth media had no significant effect on bacterial community structure, and no consistent clustering by media type was observed across ordination or hierarchical analyses. Several genera were preferentially enriched in HP systems, including *Rhodanobacter*, *Sphingomonas*, and *Pseudorhodoferax*, which collectively accounted for approximately 18–22% of the cumulative relative abundance among the top 50 genera. AQ systems exhibited higher relative abundances of genera such as *Candidatus Nitrosotalea*, *Mycobacterium*, and *Candidatus Nitrosotenuis*, together representing approximately 20–25% of dominant taxa, consistent with the distinct nutrient and microbial inputs characteristic of AQ production (Figure 6; Figure S5; Table S2). Relative abundance patterns of dominant genera further supported these system-level differences (Figure S5).

## 3. Discussion

This study shows that cucumber growth and yield were consistently higher under aquaponic (AQ) production compared with hydroponics (HP), and that this advantage was further enhanced when biochar substrates, particularly medium and coarse particle sizes, were used. Morphological traits followed a clear system trend, with AQ producing taller plants with thicker stems and longer internodes than comparable HP treatments (Table 1). Yield responses mirrored these patterns, with AQ treatments achieving the highest productivity overall and AQ coarse biochar producing the top yield among treatments (Figure 2). Prior work comparing aquaponics and hydroponics under controlled environments similarly reports that aquaponic systems can match or exceed conventional hydroponic performance depending on configuration and management, while also improving fertilizer and nutrient use efficiency in some designs [10,26,27]. System-level reviews further emphasize that nutrient delivery in aquaponics is strongly shaped by microbial mineralization and continuous organic inputs, which can stabilize nutrient availability compared with mineral-only fertigation in HP systems [6,7]. Even when EC targets are maintained within similar ranges, differences in nutrient form, timing, and microbially mediated turnover between AQ and HP systems can result in distinct plant responses, as demonstrated across multiple comparative studies [28,29,30]. Together, these reports support the interpretation that the superior performance observed under AQ in the present study likely reflects differences in nutrient form, turnover dynamics, and microbial mediation inherent to aquaponic systems, even when overall salinity targets appear similar. Therefore, results should be interpreted as system-level responses under applied greenhouse conditions rather than strictly controlled mechanistic comparisons.

Beyond cultivation system, substrate structure played a significant role in modulating crop performance, indicating that particle-size-dependent physical properties shaped the root-zone environment. Medium and coarse biochar treatments generally supported stronger plant morphology (Table 1), higher yields (Figure 2), more stable nutrient profiles (Figure 3), and improved stress balance (Figure 5) compared with coconut coir and fine biochar in several comparisons. Biochar has been widely recognized as a promising component of soilless and container substrates because it can alter bulk density, porosity, container capacity, and water-holding characteristics, pH and EC behavior, and nutrient availability, although outcomes depend strongly on biochar feedstock, production conditions, application rate, and crop species [31,32,33,34]. Biochar, as a stable substrate, can also affect nutrient retention and leaching behavior in soilless matrices; for example, biochar-amended substrates have been shown to change macronutrient leaching patterns, including nitrate, phosphate, and potassium, across sequential leaching events, demonstrating a tangible capacity to reshape nutrient loss and availability dynamics in substrate-based production [35,36]. In mechanistic terms, particle size matters because it influences packing density, pore space between particles, and how water is retained and released; controlled experiments show that biochar particle size affects water storage by modifying inter-particle pore space and contributing intrapores that alter water retention behavior [37]. These concepts align with the present findings, where medium and coarse biochar were associated with more stable nutrient dynamics, improved stress balance, and stronger canopy development.

Nutrient dynamics provided further insight into the mechanisms underlying the observed performance differences. Despite similar EC ranges across hydroponic and aquaponic systems, nutrient behavior differed markedly between systems over time, reflecting fundamental differences in nutrient delivery mechanisms. While hydroponic systems rely on externally formulated mineral solutions with relatively fixed nutrient ratios, aquaponic systems receive nutrients through continuous microbial mineralization and nitrification of fish-derived organic inputs, resulting in a dynamic and nitrate-rich nutrient supply [3,30]. In the present study, this distinction was evident in the leachate profiles, where aquaponic treatments exhibited higher mid- to late-season NO_3_–N concentrations compared with hydroponics, despite comparable EC values (Figure 3a,b). Similar system-dependent divergence was observed for K and Ca, with aquaponics maintaining higher concentrations later in the production cycle (Figure 3c,d). Notably, these elevated leachate concentrations do not indicate reduced nutrient uptake, but rather reflect greater nutrient availability and continuous replenishment that can exceed instantaneous plant demand in biologically buffered systems [38]. This interpretation is further supported by the absence of EC separation between systems (Figure 3a), indicating that differences in nutrient composition and flux, rather than total salinity, governed nutrient availability and plant response. Although nutrient targets were aligned between systems, differences in nutrient form, temporal availability, and microbial mediation likely contributed to the observed plant responses.

A key outcome of this work was that comparable EC ranges across systems did not translate into equivalent nutrient behavior or plant uptake. Studies of hydroponic nutrient solution management emphasize that EC is a useful operational indicator but cannot substitute for nutrient-specific monitoring as different ions can drift independently, and recirculating systems can exhibit nutrient imbalance even when EC remains within target ranges [39]. Consistent with that principle, leachate EC remained within a narrow band across treatments (Figure 3a), yet nutrient concentrations diverged clearly over time, with AQ tending to show higher mid to late season NO_3_–N, K, and Ca than HP (Figure 3b–d). In aquaponics, nitrate is continuously generated through microbial mineralization and nitrification of fish-derived inputs, resulting in a nitrate-rich nutrient solution whose composition differs fundamentally from mineral hydroponic fertigation even at similar EC values [3,30]. Consequently, elevated leachate NO_3_–N under aquaponics reflects greater nutrient availability and system buffering rather than reduced plant uptake [38]. This interpretation is supported by leaf tissue analyses showing higher N and K concentrations in aquaponic treatments, particularly when combined medium and coarse biochar were used (Figure 4a,b), consistent with enhanced nitrogen assimilation and tissue accumulation under aquaponic conditions despite higher residual nitrate in the leachate [40]. Calcium partitioning followed expected plant physiological patterns, with basal leaves accumulating more Ca than top leaves across treatments (Figure 4c), reflecting calcium’s limited phloem mobility and progressive deposition in older tissues [41].

The contribution of biochar to nutrient behavior is plausibly linked to its exchange and adsorption capacity and its influence on nutrient retention near the root zone. Work in soilless and substrate-based systems demonstrates that biochar can measurably alter macronutrient leaching behavior, supporting the interpretation that biochar can help buffer nutrient availability under fertigated conditions [34,37]. Broader synthesis papers further highlight biochar’s effects on chemical properties relevant to nutrient retention, including surface area development, charge characteristics, and cation exchange capacity, as well as its potential to stabilize nutrient supply under controlled-environment production, although outcomes vary with biochar type and system conditions [31,36,42]. In aquaponics specifically, system-level studies and reviews emphasize that nutrient timing and form are strongly affected by microbial processing of organic inputs and biofiltration, which can shift nutrient availability relative to mineral-salt hydroponics even at similar EC [5,6]. While biologically active root-zone environments may enhance nutrient acquisition, no significant substrate effect on overall bacterial community composition was detected. Therefore, the improved plant performance is more directly attributed to differences between hydroponic and aquaponic systems in nutrient dynamics and root-zone conditions rather than substrate-driven microbial shifts. Taken together, the present nutrient results reinforce that managing greenhouse systems using EC alone can obscure meaningful differences in nutrient profiles and uptake, and that combining nutrient-specific monitoring with deliberate substrate selection can improve predictability and performance across both HP and AQ production. Although the present results clearly showed particle-size-related differences in plant performance, additional physicochemical characterization of the biochar substrates, including bulk density, porosity, and water-holding capacity, would further strengthen the mechanistic interpretation of these responses.

Physiological responses assessed through electrophysiological monitoring further supported these interpretations [43]. Clear differences in stress exposure were observed across cultivation systems and growing media, with hydroponic coconut coir and fine biochar treatments experiencing higher proportions of the season under mild-to-high stress categories. In contrast, medium and coarse biochar treatments, particularly under aquaponic conditions, showed reduced exposure to low Plant Balance Index (PBI) ranges and more stable physiological profiles (Figure 5). Plants under the AQ system exhibited overall lower stress levels, with AQ medium and AQ coarse biochar displaying the most favorable distribution across PBI categories (Figure 5). These stress profiles closely aligned with growth and yield outcomes (Table 1; Figure 2) and were coherent with observed nutrient patterns (Figure 3 and Figure 4), suggesting that improved physiological stability may arise from the combined effects of favorable root-zone physical conditions and biologically buffered nutrient delivery. Importantly, continuous electrophysiological monitoring provided a high-resolution perspective on plant responses to environmental and root-zone fluctuations that may not be captured by periodic growth or nutrient measurements alone [44]. In applied greenhouse contexts, integrating such physiological signals into irrigation and fertigation decision-making may support earlier stress detection and improved operational resilience. Together, these results indicate that nutrient dynamics, plant growth, and physiological stability were closely linked under the different cultivation systems evaluated.

Patterns observed in microbial community composition further reinforced the dominant role of cultivation system. Aquaponic and hydroponic samples formed distinct clusters in both hierarchical clustering and principal coordinate analysis (PCoA) (Figure 6). PERMANOVA indicated that production system explained 19.3% of the observed variation in bacterial community composition, representing a moderate but statistically significant system-level effect. In contrast, growing media, including biochar particle size, did not significantly alter overall bacterial community structure, and no consistent clustering by media was evident (Figure 6). Several genera were preferentially associated with each system, with *Rhodanobacter*, *Sphingomonas*, and *Pseudorhodoferax* enriched in HP systems and *Aquicella*, *Mycobacterium*, and *Candidatus Nitrosotenuis* enriched in AQ systems (Figure 6; Supplementary Table S2). These system-specific assemblages are consistent with the expectation that aquaponics introduces continuous microbial inoculation and organic substrates via aquaculture effluent, whereas hydroponics represents a more chemically defined environment shaped by mineral salts and line or reservoir microbiota [38,40,45,46,47]. While substrate particle size did not drive whole-community separation, the physical architecture of biochar may still influence microbial microhabitats at finer spatial scales without producing effects large enough to be detected at the community level [21]. Also, PGPR can have a significant effect on plant performance in a biologically active environment, improving growth [48]. Future studies incorporating functional profiling, increased replication for microbiome endpoints, or time-resolved sampling may help clarify whether substrate-related effects emerge transiently or primarily at functional rather than compositional levels.

## 4. Materials and Methods

### 4.1. Experimental Site and Conditions

The experiment was conducted from 1 October 2024 to 29 February 2025, at the Center for Sustainable Food Production (CSFP), Lethbridge Polytechnic, Alberta, Canada (49°39′33.9″ N, 112°48′24.0″ W). The greenhouse facility (7500 ft^2^) was equipped with a recirculating irrigation system and full climate-control infrastructure (Figure S1). Prior to the experiment, the greenhouse and irrigation lines were sanitized with SaniDate^®^ 5.0 (5% peroxyacetic acid) and 1.0% hydrogen peroxide, applied at recommended concentrations and contact times, to minimize microbial contamination. Environmental conditions were maintained at 23 ± 2 °C during the day and 21 ± 2 °C at night, with relative humidity between 65–75% and vapor pressure deficit (VPD) controlled between 0.8–1.2 kPa. The experiment was conducted under natural light conditions, and no supplemental lighting was applied. An automated climate and irrigation control system (Microclimates Inc., Seattle, WA, USA) monitored and regulated climate and irrigation conditions continuously. Circulation fans were installed at the corners of each plot to ensure uniform airflow and reduce localized heat accumulation.

### 4.2. Experimental Design and Treatments

A split-plot design was implemented to evaluate the effects of cultivation system and substrate type on cucumber (*Cucumis sativus* L. cv. Verdon RZ F1) performance. The main plot factor was cultivation system (hydroponics or aquaponics). The subplot factor was substrate, consisting of three particle-size grades of 100% bamboo-derived biochar: fine (1–3 mm), medium (3–6 mm), and coarse (6–10 mm), along with coconut coir as a commercial control (Figure 1). Each main plot measured 14.52 m^2^ (3.3 × 4.4 m) and contained 24 cucumber plants, while each subplot (3.3 × 1.1 m) contained six plants, giving a plant density of 1.65 plants per m^−2^. The experiment was replicated three times, with cultivation systems randomized across blocks (Figure S3). All treatments were exposed to the same environmental and light conditions throughout the experiment to minimize confounding effects associated with spatial or microclimatic variability.

### 4.3. Cultivation Systems

The hydroponic system consisted of a closed-loop recirculating fertigation setup in which plants received a mineral nutrient solution prepared from concentrated A and B stock solutions (Figure S2; Table S1). The solution was maintained at an electrical conductivity (EC) of 1800 ± 50 µS cm^−1^ and a pH of 5.8–6.2, adjusted with 3.65% hydrochloric acid.

The aquaponic system operated as a coupled recirculating system, in which water continuously circulated between fish tanks, a multi-stage solids management and filtration system, and plant growing beds (Figure S4). Tilapia (*Oreochromis niloticus*) were stocked in recirculating aquaculture tanks (three tanks of 3000 L and four tanks of 4800 L) at a density of 50–60 kg m^−3^. Water from the fish tanks was first directed through a mechanical filtration unit consisting of a rotating drum filter fitted with a 30 µm screen to remove suspended solids. Separated solids were then directed to a settling tank and bioreactor for further processing. The clarified water subsequently passed through a biological filtration unit, including a biochar-based biofilter (28.4 m^2^; 6400 L; 8% of total system volume), to facilitate nitrification and stabilize water chemistry before being delivered to the plant beds.

The aquaculture component operated at a recirculation frequency of 1–2 tank turnovers per hour, while the overall system volume (~80,000 L) achieved a turnover rate of approximately 1.62–2.16 times per day with a leaching fraction of about 30%.

Water quality in the aquaponic system was monitored weekly, including EC (maintained near 1800 ± 50 µS cm^−1^), pH, dissolved oxygen, total ammonia nitrogen (TAN), nitrite, and nitrate. The pH was maintained at 5.8–6.2 using potassium bicarbonate. Iron and calcium were supplemented as needed, based on twice-weekly monitoring, to partially align key nutrient concentrations with the target ranges used in the hydroponic system (Table S2).

The proximate and elemental composition of the fish feed used in the aquaponic system is provided in Table S3, and a summary of fish biomass, feeding rate, growth, and mortality during the study period is presented in Table S4.

To enhance comparability between systems, irrigation frequency and duration were standardized across treatments. However, as hydroponic and aquaponic systems differ inherently in nutrient origin, microbial inputs, and internal bioprocesses, the study was designed as a comparative system-level evaluation under controlled greenhouse conditions rather than as a strictly nutrient-isolated mechanistic comparison.

### 4.4. Substrate Preparation and Irrigation Regime

Each media bag was filled with 4 kg of substrate (bamboo-derived biochar of fine, medium, or coarse grade, or coconut coir). Two drippers, each with flow rate of 1 L h^−1^, were installed per bag at 50 cm spacing. Cucumber seedlings at the three-true-leaf stage were transplanted into the media beds. Irrigation was scheduled for 30 s every 2 min during the daytime. And 1 min every 30 min during nighttime. Under this regime, total daily irrigation input was approximately 9.4 L slab^−1^ day^−1^ (equivalent to 4.7 L plant^−1^ day^−1^), with an average leaching fraction of ~30% maintained across treatments. Nutrient solutions were prepared by mixing concentrated stock solutions (A and B) into an 800 L reservoir, with pH adjusted using 3.65% hydrochloric acid. Electrical conductivity (EC) was maintained at 1800 ± 50 µS cm^−1^ and pH at 5.7–5.9 throughout the trial. Detailed nutrient solution compositions for both HP and AQ systems are provided in Supplementary Tables S1 and S2.

### 4.5. Morphological and Physiological Measurements

Plant morphological parameters, including stem elongation, stem diameter, and internode distance, were measured biweekly. The date of first flowering was also recorded for all plants within each treatment.

### 4.6. Yield and Fruit Quality Measurements

Cucumber fruits were harvested twice a week when their minimum diameter reached 41 mm. Fruits were assessed for market quality based on Canadian Food Inspection Agency (CFIA) standards [49]. Fruits were categorized into commercial grades (Large, Medium, and Small) based on diameter and weight thresholds. Total yield was calculated on a per-square-meter basis (kg m^−2^) to facilitate treatment comparisons.

### 4.7. Nutrient Analysis

Irrigation leachate was collected weekly and leaf tissues biweekly from the sixth fully expanded leaf (top canopy) and basal leaves, starting 10 days after transplantation. Leachate was analyzed for volume, EC, pH, and nutrient concentrations following AOAC guidelines [AOAC, 2016]. Leaf samples were oven-dried (65 °C), ground, and digested in trace-metal grade nitric acid using a microwave-assisted digestion system according to [50]. Macronutrients (N, P in the form of TON and phosphate) were quantified using Gallery Plus spectrophotometer (Thermo Fisher Scientific, Waltham, MA, USA) whereas K, Ca, Mg, and micronutrients (Fe, Mn, Zn, Cu, B) were measured using iCAP RQ ICP-MS (Thermo Scientific) [50].

### 4.8. Plant Stress Response Measurements

Electrophysiology recordings were conducted continuously throughout the study to capture diurnal variations in the electrical activity of plants. Eight plants from the center of each plot were selected for analysis from each treatment group. Electrical signals generated by plants were recorded using PhytlSigns devices from Vivent SA (Crans-près-Celigny, Switzerland), as described by Tran et al. [43]. For each plant, the PhytlSigns device measured the electrical potential difference between the main stem and the petiole of the 7th leaf from the base of the plant [8]. The electrical signal was sampled at a rate of 256 Hz and filtered to remove the frequencies at 60 and 120 Hz. The signal was recorded in millivolts (mV) and was captured using MATLAB software (V. R2023a) [51].

For PBI, a comparative analysis was performed by aligning each day’s signal pattern with the standard deviation of the previous four days. Instances where the recorded signal exceeded the standard deviation were marked as significant deviations, indicating plant responses to environmental changes. The PBI was calculated as a normalized ratio between these deviations and the standard deviation itself, averaged across all plants in a treatment group. PBI scores were categorized as follows: a range of 0.8–1.0 indicated marginal environmental influence and stable ambient conditions; a range of 0.4–0.8 reflected moderate adaptation to environmental factors; and a range of 0–0.4 suggested significant environmental influence and potential plant stress.

### 4.9. Microbial Community Analysis

For microbial analysis, root-zone substrate samples were collected at harvest from each treatment replicate. Samples were taken from the center of each media bag, approximately 10–15 cm below the surface, using sterile spatulas to avoid edge effects and external contamination. For each treatment, three subsamples (10 g each) were collected from different plants within the same subplot, pooled into a composite sample, and homogenized. Composite samples were placed into sterile 50 mL Falcon tubes, immediately flash-frozen in liquid nitrogen, and stored at −80 °C until further processing. Because root-zone substrate sampling was conducted only at harvest, the microbial results are interpreted as an endpoint assessment and do not capture temporal shifts in community composition during the production cycle.

DNA was extracted using the DNeasy PowerSoil Kit (Qiagen, Hilden, Germany) following the manufacturer’s protocol. The V4 region of the 16S rRNA gene was amplified using the universal primers 515F and 806R [52]. Amplicons were sequenced on an Illumina MiSeq platform (2 × 250 bp paired-end reads). Reads were demultiplexed, trimmed, denoised, and assigned to amplicon sequence variants (ASVs) with DADA2 [53]. Taxonomic assignment was conducted against the SILVA database (v138) [54]. Microbial diversity was evaluated through alpha diversity indices (Shannon, Simpson), beta diversity metrics (Bray–Curtis dissimilarity, principal coordinate analysis), and differential abundance testing using DESeq2 [55].

### 4.10. Statistical Analysis

All data were analyzed using R version 4.3.1 [56]. Data were analyzed using linear mixed-effects models. Cultivation system, growing media, and their interaction were treated as fixed effects, while block and the block × cultivation system interaction were included as random effects. Model assumptions of normality and homogeneity of variance were evaluated using the Shapiro–Wilk and Levene’s tests, respectively. When significant effects were detected, mean separation was performed using Tukey’s Honest Significant Difference (HSD) test at *p* < 0.05. Microbial community analyses were conducted separately using alpha-diversity indices, Bray–Curtis dissimilarity, principal coordinate analysis (PCoA), and permutational multivariate analysis of variance (PERMANOVA), while differentially abundant taxa were identified using DESeq2 [55] with significance evaluated at *p* < 0.05. All data are presented as mean ± standard deviation (SD), and figures were generated using ggplot2 in R.

## 5. Conclusions

Under the greenhouse conditions evaluated, cultivation system was the dominant factor shaping cucumber growth, yield, nutrient behavior, physiological stability, and root-zone bacterial community structure, while substrate particle size acted as an important secondary factor influencing plant performance. Aquaponic production consistently outperformed hydroponics despite similar EC ranges, indicating that nutrient form, temporal availability, and biologically mediated nutrient turnover played a greater role than bulk salinity alone. Among substrates, medium (3–6 mm) and coarse (6–10 mm) biochar supported improved yield performance, more stable nutrient dynamics, and reduced physiological stress compared with fine biochar and coconut coir. These findings suggest that combining aquaponic production with medium to coarse bamboo-based biochar represents a promising strategy for sustainable greenhouse cucumber cultivation. More broadly, the results highlight the importance of integrating cultivation system design, substrate physical properties, and nutrient-specific monitoring to improve crop performance in controlled environment agriculture.

## Figures and Tables

**Table 1:** Effects of irrigation system and growing medium on stem elongation, stem diameter, and internode distance.

Cultivation System	Growing Medium	Stem Elongation (cm)	Stem Diameter (mm)	Internode Distance (cm)
		Mean ± Std	Mean ± Std	Mean ± Std
Hydroponics	Coconut Coir	985.8 ± 35.4 a	13.4 ± 0.8 a	9.4 ± 0.5 a
	Coarse Biochar	1020.3 ± 38.2 a	14.1 ± 0.9 a	9.7 ± 0.4 a
	Medium Biochar	1044.7 ± 39.1 a	14.5 ± 1.0 a	10.1 ± 0.5 a
	Fine Biochar	1002.1 ± 34.8 a	13.9 ± 0.7 a	9.8 ± 0.4 a
Aquaponics	Coconut Coir	1058.6 ± 37.6 ab	13.8 ± 0.8 a	9.9 ± 0.5 ab
	Coarse Biochar	1076.5 ± 39.3 b	14.8 ± 0.9 a	10.3 ± 0.5 b
	Medium Biochar	1102.0 ± 40.1 b	15.1 ± 1.0 a	10.6 ± 0.6 b
	Fine Biochar	1082.4 ± 38.5 b	14.5 ± 0.8 a	10.2 ± 0.4 b

Values represent mean ± standard deviation. Different lowercase letters within each column indicate statistically significant differences among treatments according to Tukey’s HSD test (*p* < 0.05).

## Data Availability

The raw data used to analyze for this study are available by contacting the corresponding authors.

## Supplementary Materials

The following supporting information can be downloaded at: https://www.mdpi.com/article/10.3390/plants15111627/s1. Figure S1: Layout of controlled environment agriculture facility used in the experiment.; Figure S2: Schematic diagram of circulating hydroponics system used in this experiment.; Figure S3: Schematic of experimental designs.; Figure S4: Schematic layout of the recirculating aquaculture system used in this study (total area: 610.10 m²; scale 1:50).; Figure S5: Relative abundance of the top 20 bacterial genera in hydroponic (HP) and aquaponic (AQ) systems.; Table S1: Fertilizer recipe for stock solutions A and B.; Table S2: Elemental composition of the feeding solutions supplied to the hydroponic and aquaponic systems.; Table S3: Proximate and elemental composition of the fish feed used in the aquaponic system.; Table S4: Summary of aquaculture system performance during the experimental period.; Table S5: Mean leaf tissue nutrient concentrations (±SD) and statistical groupings for hydroponic (HP) and aquaponic (AQ) treatments with different growing media and leaf positions.

## Abbreviations

The following abbreviations are used in this manuscript:

AQ	Aquaponic system
HP	Hydroponic system
CEA	Controlled Environment Agriculture
EC	Electrical Conductivity
PBI	Plant Balance Index
NO_3_–N	Nitrate nitrogen
K	Potassium
Ca	Calcium
LAI	Leaf Area Index
PCoA	Principal Coordinate Analysis
PERMANOVA	Permutational Multivariate Analysis of Variance
ICP-MS	Inductively Coupled Plasma Mass Spectrometry
16S rRNA	16S ribosomal RNA gene
DNA	Deoxyribonucleic Acid
