# Supplementary material for: Cultivation System Dominates Cucumber Performance and Root-Zone Microbiomes Across Biochar Particle Sizes
Source: Plants (Basel). 2026 May 26;15(11):1627. doi: 10.3390/plants15111627 (PMC13259428; doi:10.3390/plants15111627)
Supplement: Supplementary file 1 [file plants-15-01627-s001.zip › plants-4233192-supplementary.pdf]

Supplementary Material

Supplementary Figures:

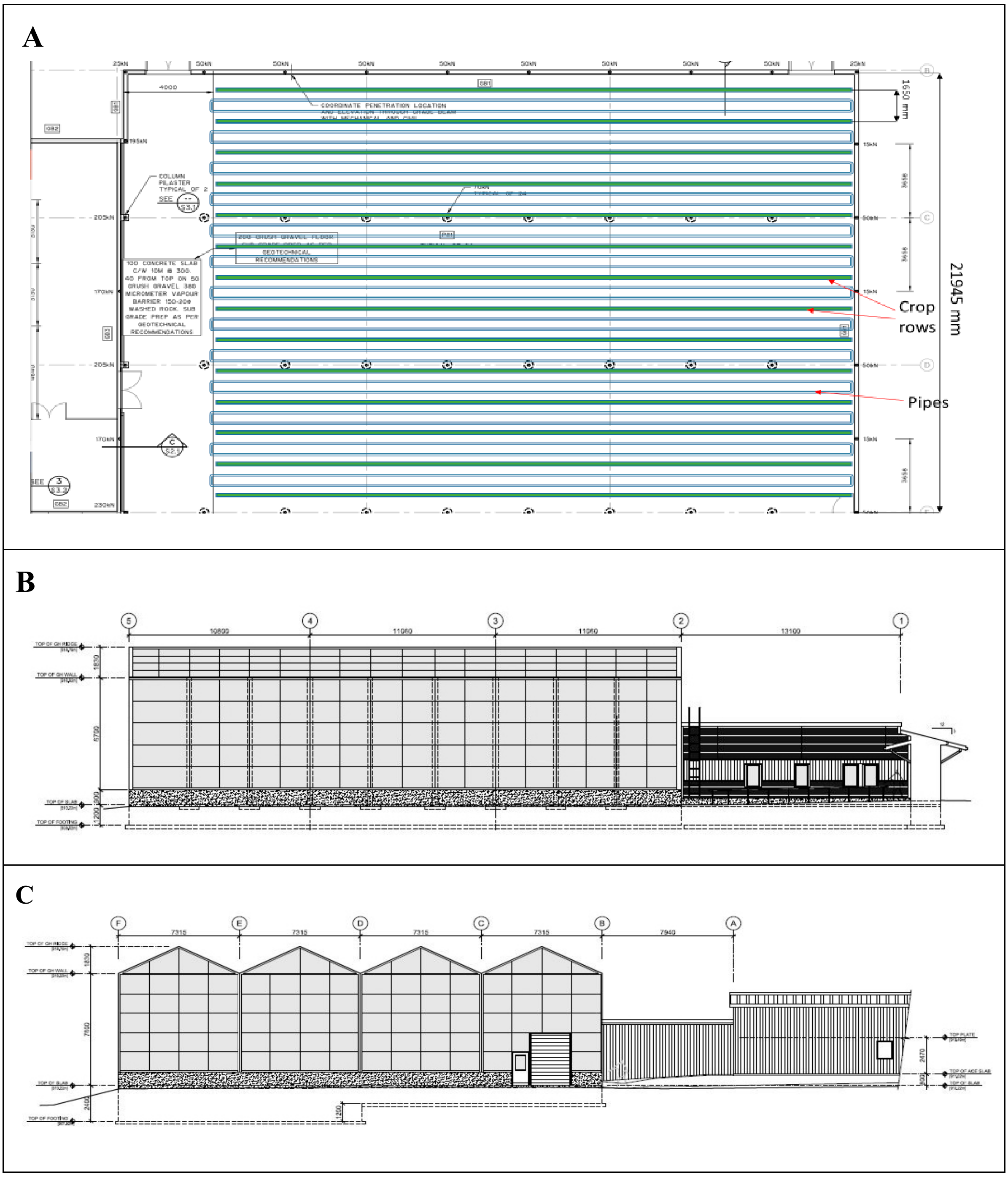

Figure S1. Layout of controlled environment agriculture facility used in the experiment. Panel A shows the top-down view of the facility layout, indicating the arrangement of crop rows and irrigation pipes with detailed measurements. Panels B and C provide the elevation views of the facility; Panel B illustrates the side elevation, and Panel C shows the front elevation, highlighting the structural design and dimensions of the greenhouse and associated buildings.

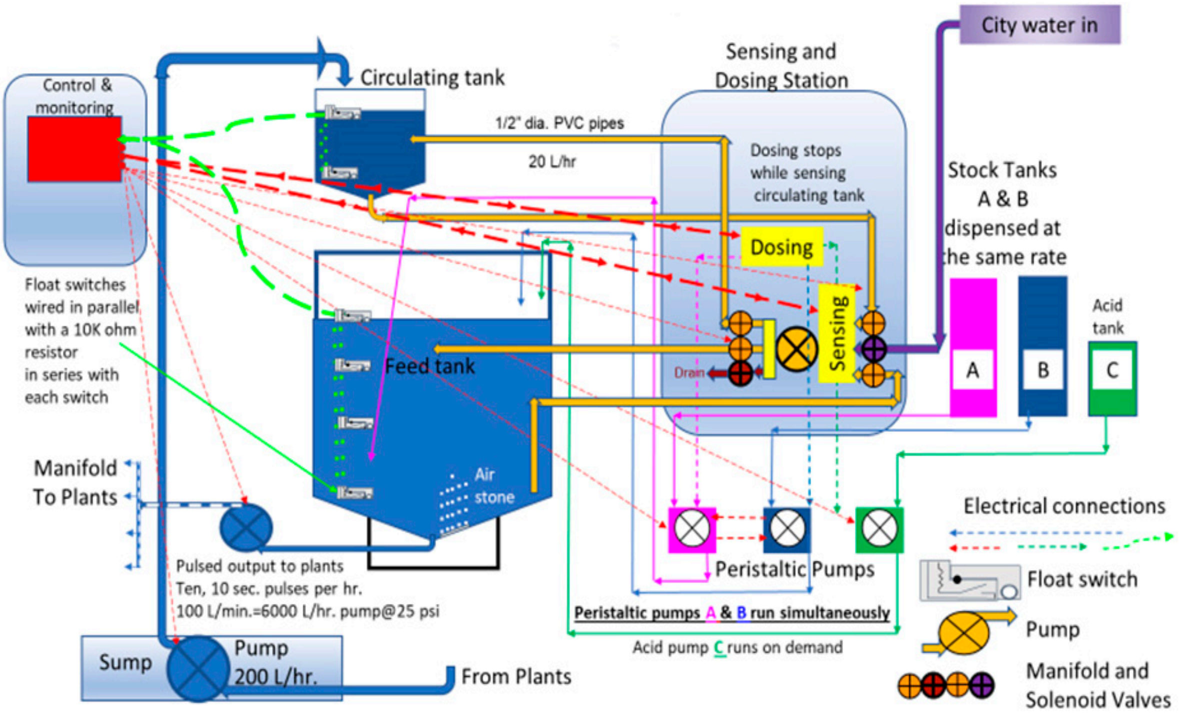

Figure S2. Schematic diagram of circulating hydroponics system used in this experiment. This diagram illustrates the components and flow of the system. It includes control and monitoring units, circulating and feed tanks, a sensing and dosing station, and stock tanks for nutrient solutions and acid. Peristaltic pumps are used to dispense nutrients, while a main pump circulates the solution through the manifold to the plants. The sump collects runoff, which recirculates in turn. Float switches and electrical connections ensure automated control and monitoring of the nutrient delivery process.

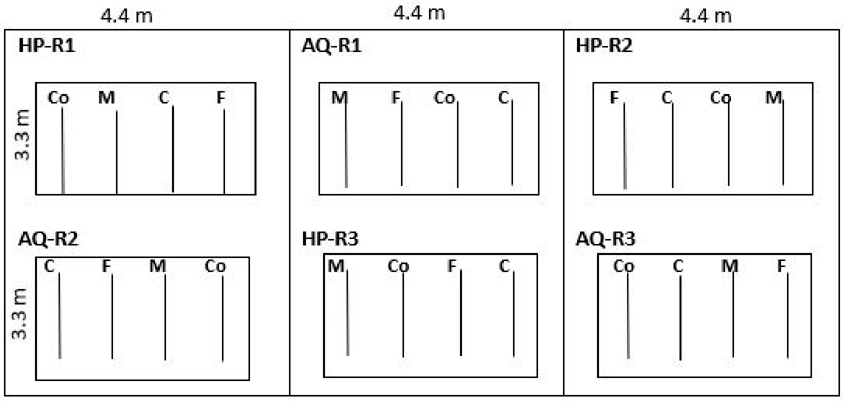

Figure S3. Schematic of experimental designs. Schematic diagram of the split-plot experimental design used to evaluate the effects of cultivation systems and growing medium on cucumber performance under greenhouse conditions. The main plots represent two cultivation systems: hydroponics (HP) and aquaponics (AQ), each with three replications (R1–R3). Within each main plot, subplots represent the four growing media: Co = Coconut coir (control), F = Fine-grade biochar, M = Medium-grade biochar, and C = Coarse-grade biochar.

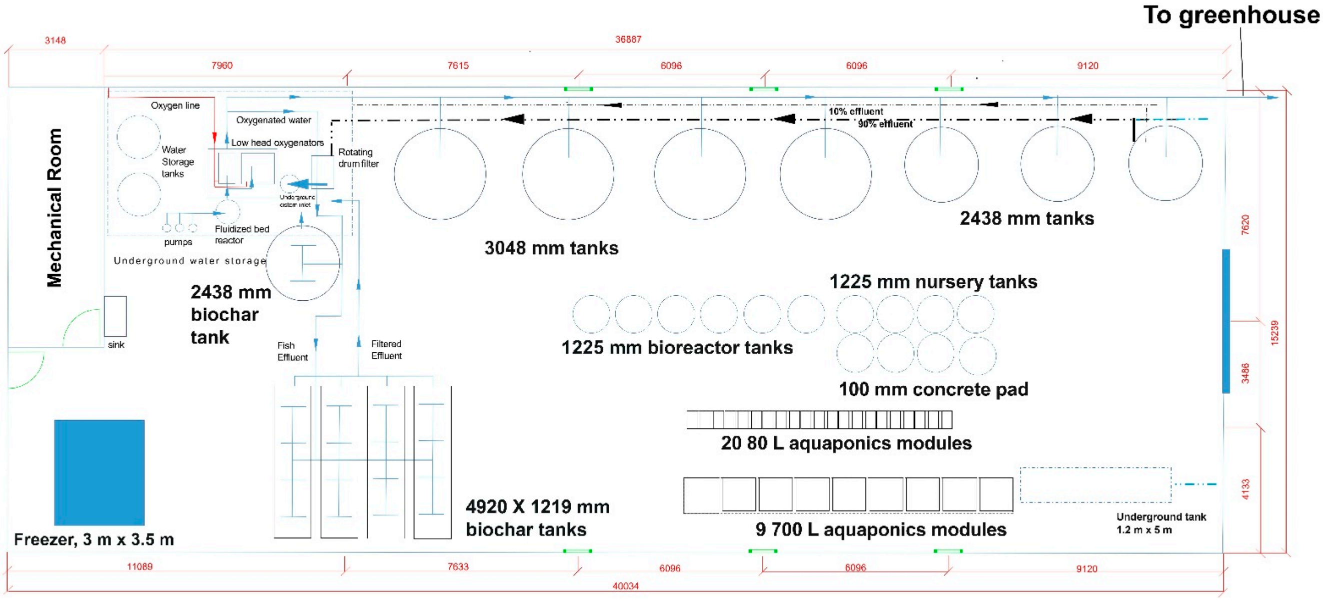

Figure S4. Schematic layout of the recirculating aquaculture system used in this study (total area: 610.10 m²; scale 1:50). The system included fish tanks, nursery tanks, biochar filtration tanks, water storage units, pumps, low-head oxygenators, a rotating drum filter and a fluidized bed reactor located adjacent to the mechanical room. Fish effluent was mechanically and biologically filtered, with 90% recirculated and 10% discharged.

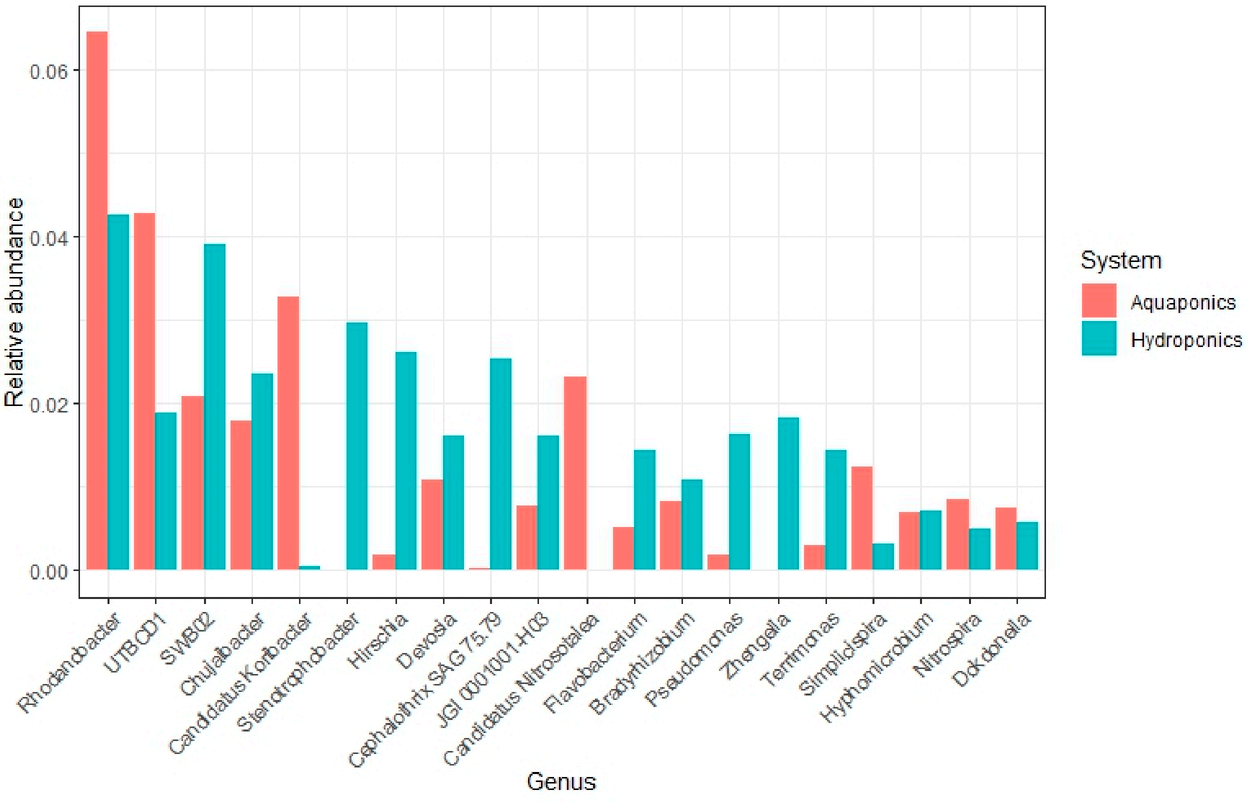

Figure S5. Relative abundance of the top 20 bacterial genera in hydroponic (HP) and aquaponic (AQ) systems.

1 Supplementary Tables:

Table S1. Fertilizer recipe for stock solutions A and B.

STOCK-A	Amount of fertilizer (PPM)
Potassium nitrate 46.3%-13.7%	35000
Calcium nitrate 18.8%-15.5%	85000
Iron chelate (EDTA) 13.2%	3000
**STOCK-B**	**Amount of fertilizer (PPM)**
Potassium nitrate 46.3%-13.8%	35000
MKP 28.15% - 22.44%	19000
Magnesium sulphate 12%-9.8%	40000
Manganese chelate 13%	360
Zinc chelate 14%	135
Cooper chelate 14%	135
Borax 11%	300
Sodium (molybdate) 39.7%	10

Table S2. Elemental composition of the feeding solutions supplied to the hydroponic and aquaponic systems in this study (±5% variation), standardized to an electrical conductivity (EC) of 1800 ± 50 µS/cm.

Element	Hydroponics Feeding Solution (PPM)	Aquaponics Feeding Solution (PPM)
N	168	165
P	33	36
K	217	222
Ca	134	142
Mg	36	32
S	41	101
Fe	3	0.3
Mn	0.36	0.39
Zn	0.14	0.13
B	0.27	0.06
Cu	0.14	0.27
Mo	0.03	0.001

Table S3. Proximate and elemental composition of the fish feed used in the aquaponic system.

Parameter	Value	Unit
Crude protein	40	%
Crude fat	9	%
Crude fiber	2	%
Boron (B)	0.0153	mg g^{-1} DW
Sodium (Na)	3.73	mg g^{-1} DW
Magnesium (Mg)	5.28	mg g^{-1} DW
Potassium (K)	9.91	mg g^{-1} DW
Calcium (Ca)	9.5	mg g^{-1} DW
Manganese (Mn)	0.0856	mg g^{-1} DW
Iron (Fe)	0.233	mg g^{-1} DW
Copper (Cu)	0.00368	mg g^{-1} DW
Zinc (Zn)	0.0342	mg g^{-1} DW
Molybdenum (Mo)	0.0088	mg g^{-1} DW
Phosphorus (P)	10.53	mg g^{-1} DW
Nitrogen (N)	64.48	mg g^{-1} DW

*DW: dry weight

Table S4. Summary of aquaculture system performance during the experimental period.

Parameter	Value / Range	Description
Fish population	2400 fish	Mixed cohorts (grow-out, large, and small fish)
Stocking density	45–55 kg m^{-3}	Across recirculating tanks
Feed rate	0.9–1.0% biomass day^{-1}	Reduced during holidays and operational constraints
Feed conversion ratio (FCR)	~1.25	Increased during reduced feeding and system interruptions
Growth rate	~0.15–1.1% day^{-1}	Varied by cohort and management conditions
Mortality rate	4–5%	Within acceptable operational range
Biomass estimation	Every 2–3 weeks	Based on periodic weight measurements

*Values are presented as approximate ranges due to system variability are intended to characterize nutrient input rather than aquaculture production performance.

Table S5. Mean leaf tissue nutrient concentrations (± SD) and statistical groupings for hydroponic (HP) and aquaponic (AQ) treatments with different growing media and leaf positions.

Cultivation System	Growing Media	Leaf Position	N (% DW)	Group	K (% DW)	Group	Ca (% DW)	Group
HP	Coir	Top	3.67 ± 0.20	d	3.08 ± 0.20	e	1.83 ± 0.23	d
HP	Coir	Basal	3.60 ± 0.18	d	3.05 ± 0.23	e	2.19 ± 0.17	c
HP	Biochar F	Top	3.80 ± 0.22	c	3.22 ± 0.25	de	1.97 ± 0.19	cd
HP	Biochar F	Basal	3.64 ± 0.18	c	3.19 ± 0.29	de	2.32 ± 0.17	bc
HP	Biochar M	Top	3.79 ± 0.23	c	3.44 ± 0.24	bc	2.05 ± 0.16	cd
HP	Biochar M	Basal	3.62 ± 0.22	c	3.33 ± 0.21	cd	2.32 ± 0.21	bc
HP	Biochar C	Top	3.87 ± 0.21	bc	3.42 ± 0.25	bc	2.08 ± 0.21	cd
HP	Biochar C	Basal	3.66 ± 0.15	c	3.37 ± 0.22	cd	2.38 ± 0.17	b
AQ	Coir	Top	3.97 ± 0.20	b	3.42 ± 0.33	bc	1.89 ± 0.21	d
AQ	Coir	Basal	3.67 ± 0.20	c	3.36 ± 0.29	cd	2.33 ± 0.20	bc
AQ	Biochar F	Top	4.08 ± 0.17	a	3.50 ± 0.23	abc	1.98 ± 0.21	cd
AQ	Biochar F	Basal	3.81 ± 0.20	b	3.43 ± 0.21	bc	2.32 ± 0.16	bc
AQ	Biochar M	Top	4.10 ± 0.20	a	3.63 ± 0.19	ab	2.05 ± 0.18	cd
AQ	Biochar M	Basal	3.92 ± 0.24	ab	3.45 ± 0.21	bc	2.53 ± 0.21	a
AQ	Biochar C	Top	3.98 ± 0.28	ab	3.64 ± 0.16	a	2.09 ± 0.25	cd
AQ	Biochar C	Basal	3.93 ± 0.29	ab	3.51 ± 0.26	abc	2.47 ± 0.18	ab

Grouping letters were derived from Tukey's HSD post hoc comparisons including cultivation system, growing media, and leaf position. Different letters indicate significant differences at $p < 0.05$.
